# Risk of infection in primary, elective total hip arthroplasty with direct anterior approach or lateral transgluteal approach: a prospective cohort study of 1104 hips

**DOI:** 10.1186/s12891-016-1332-0

**Published:** 2016-11-14

**Authors:** Thomas Ilchmann, Werner Zimmerli, Lilianna Bolliger, Peter Graber, Martin Clauss

**Affiliations:** 1 Interdisciplinary Unit for Orthopedic Infections, Kantonsspital Baselland, Rheinstrasse 26, 4410 Liestal, Switzerland; 2 Leonardo, Hirslanden Klinik Birshof, Reinacherstrasse 28, 4142 Münchenstein, Switzerland; 3 Department for Orthopedics and Trauma Surgery, Kantonsspital Baselland, Rheinstrasse 26, 4410 Liestal, Switzerland

**Keywords:** PJI, Direct anterior approach, Lateral transgluteal approach, Total hip arthroplasty, Prosthetic joint infection

## Abstract

**Background:**

The direct anterior approach (DAA) is increasingly popular for hip replacement. However, the small incision and the location near to the groin might increase the risk of periprosthetic joint infection (PJI). We asked the questions (i) whether there is an increased risk of infection for this approach, and (ii) whether the spectrum of microorganisms differs between patients with DAA and those with lateral transgluteal approach (LAT).

**Methods:**

All patients operated between 08/2006 and 12/2013 were followed prospectively in an in house register. The DAA was introduced as routine in 02/2009 at our hospital. Patients with primary elective hip replacement without previous operations were included. Follow-up was scheduled after 6, 12 weeks and 1, 2 years. PJI was defined according to standardized criteria.

**Results:**

One thousand one hundred four patients were studied, 700 were operated with DAA and 404 with LAT. No patient was lost to follow-up. PJI was diagnosed in 23/1104 (2.1 %) patients, 16 (2.3 %) in the group with DAA, and 7 (1.7 %) in the group with LAT. Patients with infection had a higher BMI (*p* < 0.001) and a higher ASA score (*p* < 0.001). Only patients with the DAA had exogenous PJI caused by gramnegative bacilli (35.7 % vs 0 %, *p* = 0.26). In the DAA-group, the fraction of patients with polymicrobial infection was somewhat higher than in the LAT-group (50 % vs 33 %, *P* = 0.64).

**Conclusion:**

There was no increased risk of infection for the DAA.

## Background

Periprosthetic joint infection (PJI) is one of the major complications after total hip arthroplasty (THA), with a reported incidence of 0.5 to 3 % [1]. Many factors contribute to the risk of PJI, some of them can be influenced by appropriate measures [2]. Recently, an increasing risk of PJI has been described in the Nordic hip registers [3] and from the US [4]. The reported infection rates depend on the definition of infection, the awareness of the surgeon, the quality of reporting, and the length of follow-up. In implant registers, there might be confounding factors, and data might be less reliable as compared to specific prospective infection registration [3, 5, 6].

**Fig. 1:**
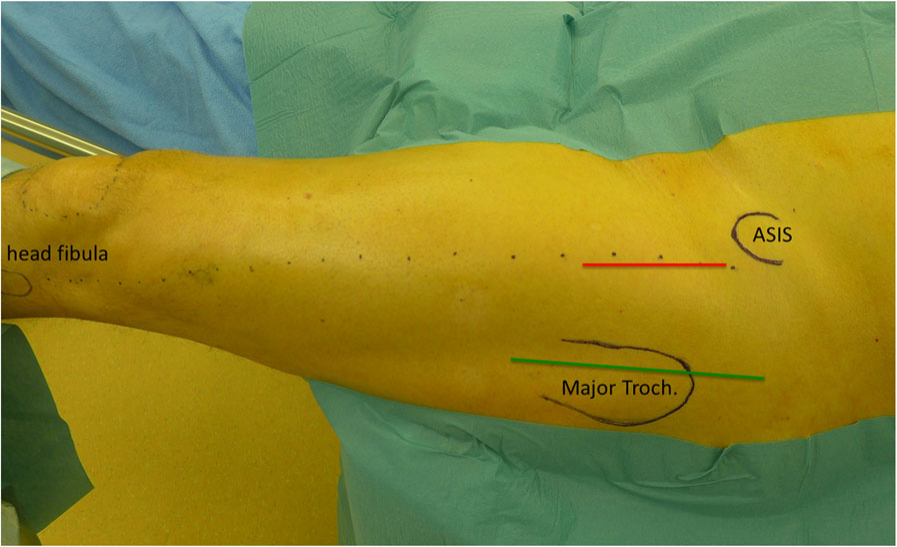
For the DAA (red line) a straight incision following the proximal part of a line (dotted) between the anterior superior iliac spine (ASIS) and the head of the fibula was made. For the LAT (green line) a straight incision was used parallel to the femur in the middle of the major trochanter

The surgical approach affects the posture of the patient, the effectiveness of laminar air flow and the draping [7]. In addition, skin quality including the type of the microbiome differs at the various incision sites [8]. Thus, the surgical approach itself might influence the rate of PJI, as well as the spectrum of infecting microorganisms. For the posterior and lateral approaches, such a difference could not be shown [9]. However, there is an increasing interest in the direct anterior approach (DAA), being assumed to be more anatomical and thus less invasive. For the DAA, the incision is closer to the groin (Fig. 1), which is a highly colonized region.

It has been shown, that groin incision is a risk factor for vascular graft associated infections [10]. Furthermore, aiming to shorter incisions might be a potential risk of increased skin irritation by traction and by surgical instruments. This might cause problems with wound healing or contamination of the implant with consecutive PJI [11–13].

In our hospital, we introduced DAA according to Matta [14] as routine in 2009 for primary elective total hip replacement [15]. Three years after introduction we observed a clustering of early infections caused either by gram-negative bacilli or by multiple microorganisms (polymicrobial). Thus, we asked the question as to whether there is (i) an increased risk of infection for DAA, and (ii) whether the spectrum of microorganisms differs between patients with DAA and those with the lateral transgluteal approach (LAT), being routine at our hospital until 2009.

## Methods

This is a retrospective observational study with prospectively acquired data of a cohort from our University affiliated academic teaching hospital with an interdisciplinary unit for orthopedic infections. This study was approved by the local Ethics Committee (Northwest and Central Switzerland, EKNZ 2015–426). In accordance with the Ethics Committee, no specific informed consent was required.

Between 08/2006 and 12/2013, 1280 primary THA were performed. Only elective hip replacements without previous hip surgery were included in the study. In total, 176 hips were excluded, 86 had a hip fracture, 78 had previous hip surgery, 11 had bone metastases, and one had rapid destructive inflammation. Out of the 1104 included hips, 700 (63 %) were operated with DAA, and 404 with LAT (Table 1).

**Table 1:** Study population

	DAA *n* = 700 (63 %)	LAT *n* = 404 (37 %)	Total *n* = 1104	*p*
Age (median, SD)	71 (10.6)	71 (±10.6)	71 (±10.6)	n.s.
Male	369 (53 %)	212 (53 %)	581 (53 %)	n.s.
BMI (mean, SD)	26.6 (4.2)	27.2 (5.2)	26.8 (4.6)	0.009
ASA I and II	575 (82 %)	338 (84 %)	913 (83 %)	n.s.
Primary osteoarthritis	644 (92 %)	375 (93 %)	1019 (92 %)	n.s.
Head necrosis	44 (6 %)	16 (4 %)	60 (5 %)	
Inflammation (RA)	7 (1 %)	2 (0.5 %)	9 (0.8 %)	
Other indications	5 (1 %)	11 (3 %)	16 (2 %)	

*DAA* direct anterior approach, *LAT* lateral approach, *BMI* body mass index (kg/m2), *ASA* American Society of Anesthesiologist, *RA* rheumatoid arthritis

The lateral approach in supine position (Fig. 1) was exclusively used until 3/2009, i.e. during the first 31 months of the study. Thereafter, the DAA was introduced. The first 100 DAA interventions were performed by 2 experienced hip surgeons. Later young consultants and residents were trained as it was standard for LAT too. After introduction of DAA, 34 hips were still operated via LAT for various reasons. Eight had previous surgery on the opposite via a lateral approach and requested the same approach. In 7 hips, DAA was not performed because of skin irritations in the groin, in 6 because of obesity, in 4 because of severe flexion contracture or a repair of the gluteal muscles, in 5 for teaching reasons, in one because of hip dysplasia and a deficient acetabular roof, and in three because no DAA trained surgeon was available.

For the DAA, the patients were postured in supine position on a trauma table [14]. We used the direct anterior approach (Fig. 1) according to Smith-Peterson [16] with a split of the fascia of the *tensor fascia lata* and preparation through the intramuscular plane between *M. sartorius* and *M. tensor fascia latae* [15]. Patients operated from lateral [17] were posed in supine position, the peri- and postoperative setting was the same.

All interventions were performed in laminar airflow without protective helmets. Patients were covered with forced air flow blankets. Single shot antibiotic prophylaxis (cefuroxime 1.5 g) was administered 30–60 minutes prior to skin incision. The skin was disinfected three times with a povidone-iodine solution (Betaseptic™, Mundipharma, Basel, CH) for totally 5 minutes [18]. Afterwards, in the DAA group, a plastic trauma draping (3 M, Rüschlikon, CH) was used, around the planned skin incision, the drape was removed and the skin was disinfected once more. The LAT group was operated without a plastic draping. Two pairs of gloves were used, before start of surgery the outer pair of gloves was changed. Perioperatively, the wound was cleaned and disinfected with a 0.1 % polyhexanide-solution (Lavasept^TM^ BBraun, Sempach, CH). The skin was closed with subcutaneous and cutaneous sutures.

Sutures were removed by the family practitioner or by the rehabilitation staff 12–14 days after surgery. Outpatient physicians were informed to resubmit the patient without prescribing antibiotics in case of any wound healing disturbance (redness, dehiscence, oozing).

The most frequently used implants were spherical pressfit cups and cemented or uncemented stems (Table 2).

**Table 2:** Type of implants

	Implant	Number	Percent
Cups	RM and RM vitamys (Mathys)	615	56 %
	Selexys TH+ (Mathys)	174	16 %
	Allofit (Zimmer)	261	24 %
	PE cem	24	2 %
	Others	30	3 %
Uncemented stems	TwinSys (Mathys)	400	36 %
	Avenir (Zimmer)	84	8 %
	CBC (Mathys)/CLS (Zimmer)	182	16 %
Cemented stems	TwinSys (Mathys)	272	36 %
	CCA (Mathys)/Muller Straight Stem	166	15 %

Body mass index (BMI), American Society of Anesthesiologist (ASA) score, procedure time and blood-loss were taken from the charts. All patients were prospectively followed in our in-house register, which has been introduced in 1984. There was an approval of the local ethical authorities for follow-up visits (EKNZ 2015–426). Follow-up was scheduled at 6 and 12 weeks, and after 1, 2 and 5 years. A standardized clinical and radiological follow-up protocol was followed, including registration of any adverse events [15]. All patients were seen at every follow-up by one out of 3 consultants of the hip unit.

PJI was diagnosed according to the IDSA-guidelines [19]. At least one of the following criteria had to be fulfilled: (a) presence of a sinus tract, (b) visible pus surrounding the joint without other explanation (e.g. no crystals), (c) acute inflammation on histopathological examination (>5 neutrophils/high-power field), (d) >4200 leukocyte per μl and/or >80 % polymorphonuclear leukocytes in synovial fluid, (e) growth of the same microorganism in at least two cultures of synovial fluid, peri-prosthetic tissue and/or sonication fluid. Patients were classified into acute postoperative (≤1 month after implantation), acute hematogenous (≤3 weeks of infectious symptoms), and chronic PJI (all other situations) [20]. As a rule, patients with PJI were managed according to an established algorithm [21] with debridement and implant retention (DAIR), one-stage or two-stage procedures, respectively [22].

### Statistics

A Shapiro-Wilk test for normality was performed to determine whether continuous data were normally distributed. All data were non-normal distributed and were presented as median and range. Mann Whitney rank sum test was used for comparison of two continuous (but non-normal distributed) variables, Chi Square or exact Fisher test for comparison of categorical variables. Conclusions about ASA Score were considered with regard to two consolidated classes: First class includes ASA Score I and II versus second class with ASA Score III and IV. A *P*-value of ≤ 0.05 was considered to be statistically significant. Data were analyzed using IBM SPSS Statistics Version 23.

## Results

The minimum follow-up was two years; no patient was lost to follow-up. None of the patients was treated with antibiotics for suspected PJI before evaluation by the interdisciplinary infection team. Of all 1104 THA, 23 (2.1 %) had PJI during the follow-up period.

Acute postoperative PJI (median 17 days, range 13 to 30 days postoperative) was diagnosed in 11 THA, 8 in the DAA- and 3 in the LAT-group. Three THA had acute hematogenous PJI (3.5, 5 and 8 months, respectively, after implantation). All THA with acute PJI were treated with DAIR, mostly with exchange of the modular parts of the device. Nine THA had chronic PJI (median 11.4, range 1.5 to 47 months after implantation). Two were treated with DAIR within the first 50 days after implantation, five were treated with one-stage exchange and two with two-stage exchange. At the final follow-up, no patient with treatment for PJI had signs of persistence of infection (Table 3).

**Table 3:** Patient characteristics, microorganisms, type of infection and surgical treatment of all episodes with PJI

UPI	Age	Gender	BMI	ASA	Approach	Microorganisms	Type of PJI^a^	Time after implantation [d]	Treatment
874	83	male	34.3	3	DAA	*S. epidermidis, Proteus mirabilis*	1	13	DAIR
873	73	female	33.9	2	DAA	*S. epidermidis, Morganella morganii Klebsiella pneumoniae, Pseudomonas aeruginosa, E. coli*	1	15	DAIR
1028	56	male	32.4	2	DAA	*S. aureus*	1	15	DAIR
987	77	male	33.8	3	DAA	*S. epidermidis, S. haemolyticus, Aerococcus* sp., *Acinebacter* sp.	1	18	DAIR
812	66	male	29.0	3	DAA	*S. epidermidis*	1	20	DAIR
709	68	female	37.7	2	DAA	*S. epidermidis*	1	21	DAIR
932	88	male	26.0	3	DAA	*S. aureus, Citrobacter freundii*	1	28	DAIR
978	71	female	36.8	3	DAA	*S. epidermidis*	1	30	DAIR
840	76	female	37.8	3	DAA	*S. epidermidis,* Gr.B *Streptococcus, Morganella morganii*	3	50	DAIR
532	83	female	32.9	3	DAA	Gr.G *Streptoccus*	2	107	DAIR
572	75	female	26.2	3	DAA	*CNS, Propionibacterium acnes*	3	129	1-stage exchange
1236	73	female	27.3	2	DAA	*S. lugdunensis*	2	160	DAIR
440	72	female	36.3	2	DAA	CNS*, S. epidermidis, Enterococcus faecalis, Propionibacterium* sp.	3	347	2-stage exchange
1162	47	female	19.1	2	DAA	*S. aureus*	3	386	1-stage exchange
835	83	male	29.6	2	DAA	*Finegoldia magna*	3	416	2-stage exchange
717	74	male	27.1	1	DAA	*Streptococcus sanguinis*	3	1431	1-stage exchange
883	63	female	45.6	2	LAT	*S. epidermidis, Enterococcus faecalis, S. warneri*	1	17	DAIR
599	84	male	33.3	3	LAT	*S. epidermidis, Bacillus* sp.	1	17	DAIR
109	73	female	29.0	2	LAT	CNS	1	17	DAIR
1121	64	male	36.6	2	LAT	*Actinomyces* sp.	3	46	DAIR
1100	65	male	40.3	3	LAT	*Streptococcus* sp.	2	238	DAIR
453	81	male	28.3	3	LAT	CNS	3	306	1-stage exchange
84	74	male	27.7	1	LAT	CNS	3	1124	1-stage exchange

*Abbreviations*: ^a^1: acute postoperative PJI, 2: acute hematogenous PJI, 3: chronic PJI, *DAIR* debridement and retention including exchange of mobile parts, *PJI* periprosthetic joint infection, *BMI* body mass index, *ASA* American Society of Anesthesiologist score, *UPI* unique patient identification number, *CNS* coagulase negative staphylococci

As we wanted to analyze, whether there is an increased risk for exogenous PJI with the DAA, we excluded the three cases with hematogenous PJI (2 DAA, 1 LAT) from further analysis. The rate of exogenous PJI was similar in both groups, namely 14/700 (2.0 %) in the DAA- and 6/404 (1.5 %) in the LAT-group (*P* = 0.54). Five of the 34 patients treated with the LAT-approach during the DAA-period suffered from a PJI; one was operated from lateral due to severe adipositas (BMI 45 kg/m2, UPI 883), three due to skin irritations in the groin making an DAA approach impossible (UPI 599, 1100, 1121), and one because no DAA trained surgeon was available (UPI 453). Table 3 summarizes patient characteristics, microorganisms, type of infection and surgical treatment of all patients with PJI. The spectrum of microorganisms was different in the two groups. Only patients with the DAA had PJI caused by gramnegative bacilli (5/14 vs 0/6, *P* = 0.26). In addition, in the DAA-group, the fraction of patients with polymicrobial infection was somewhat higher than in the LAT-group (7/14 vs 2/6, *P* = 0.64). Six out of 7 polymicrobial PJI in the DAA-group were caused either by gramnegative bacilli or *Enterococcus faecalis*, which reflects the inguinal microbiome.

Patients with PJI had a higher BMI than those without (median 33.3, range 19 to 46 vs median 26.8, range 17 to 55 vs; *P* < 0.001) and a higher ASA class (*P* < 0.001). However, they did not differ in operation time (median 116 [70 to 223] vs median 109 [44 to 287]; *P* = 0.20), gender (*P* = 0.83), age (median 74 [47 to 88] years vs median 71 [27 to 93] years; *P* = 0.24) and blood loss (median 650 [200 to 1300] ml vs median 600 [30 to 3600] ml, *P* = 0.48). Patients in the DAA group had a lower BMI (*P* = 0.01) as compared to the LAT-group (Table 1). In addition, they had less blood loss (median 500 [95 to 3600] ml vs median 700 [30 to 2800] ml, *P* < 0.001) and a shorter operating time (median 106 [44 to 236] minutes vs median 114 [49 to 287] minutes, *P* < 0.001).

## Discussion

The presence of an implant increases the risk for infection more than 100’000fold [23, 24]. Therefore, the perioperative bacterial load around the incision site is a crucial risk factor for infection. Three years after switching from LAT to DAA as the routine approach, we observed a clustering of early infections with an unusual spectrum of microorganisms. We therefore asked the questions, whether in patients undergoing THA, the DAA, being located closer to the groin, would increase the rate of PJI, and whether the spectrum of microorganisms would differ in patients with the DAA as compared to those with the LAT-approach.

The published incidence of PJI after THA is 0.5 to 3 % [1]. In patients with DAA, an incidence of up to 3 % is described [13, 25]. Because patients undergoing debridement without exchange of mobile parts, as well as those with exclusive suppressive therapy might not appear in registers, the published infection rates may be underestimated [1]. We compared the rate of PJI in infection rate in patients with DAA vs those with LAT in our cohort of patients, including all consecutive patients undergoing elective THA and any kind of infection treatment. None of the suspected early exogeneous PJI was treated as a superficial infection with antibiotics alone. In case of wound healing disturbances, PJI was actively searched with an additional debridement and microbiological sampling. Our observed infection rate seems rather high but still it was within the published range. We controlled our clinical routines and found no mitigating factors that might contribute to a higher risk of infection [7]. Furthermore the rate of implant exchange due to PJI was comparable to register data thus the used DAA generally might not be considered as risk factor for PJI [26, 27]. But larger RCT trials should be performed to compare the DAA to established lateral and posterior approaches.

In our series, 87.0 % of the patients with PJI had either acute or chronic exogenous infection independent from the approach. Thus, intraoperative contamination or implant contamination in the early postoperative time was by far the most frequent mechanism of infection. The overall infection rate was not different in the two groups. Thus, in contrast to published data [26] the introduction of the new approach was rather safe, which is probably due to the fact that the first 100 cases with the new incision site were operated by the same two experienced surgeons [15]. Exogenous polymicrobial infections were somewhat more frequent in the DAA- as compared to the LAT-group (50.0 % vs 33.3 %, *P* = 0.64), however, this difference was statistically not significant. Since in our previous cohort study of PJI from 1984–2001, the fraction of patient with polymicrobial PJI was only 12.7 % (8/63) [28] in patients treated with the LAT approach, the DAA might be a risk factor for polymicrobial PJI in a larger cohort. Polymicrobial PJI are mainly observed in patients with wound healing disturbances. Thus, these infections are acquired during the early postoperative period. The risk of polymicrobial PJI might be lowered for DAA by a more lateral skin incision keeping more distance to the groin and the use of modern dressing techniques avoiding skin folds that might cause sweating and contamination. In addition, this modified approach reduces the risk for damaging the cutaneal femoral lateral nerve too [29]. We adopted this technique during the observation period after observing a clustering of infections and felt that there was no persistence of that problem.

The mean BMI and ASA score of the studied patients did not differ from published populations, indicating that our cohort represents a normal population of THA patients. As in previously published studies, patients with a higher BMI, a higher ASA score and longer operating time had a significantly increased risk of PJI in our entire cohort. These patients are at risk irrespective of the incision site [12, 13].

The main limitation of our study is the lack of randomization concerning the approach. A possible selection bias was introduced by operating 34 patients with the lateral approach, during the period when we already switched to the DAA. Five of the patients treated with the LAT-approach during the DAA-period suffered from PJI, three of them had skin irritations in the groin making DAA not suitable. This might be confounding, and it cannot be excluded that the infection rate of the DAA group might have been higher, if all consecutive patients were operated with the DAA. An additional limitation is the relatively low number of patients, as compared to register studies with several thousands of patients. Thus, the study is likely to be underpowered leading to a lack of significant differences. The increased risk for gramnegative and polymicrobial infection should be verified, when data from larger cohorts will be available. At the present time, such data are missing, since minimal invasive approaches have been popularized only recently in Europe and the US. Strengths of our study are the complete follow-up of all patients, the completeness of data on ASA, BMI and causative microorganisms and the absence of undiagnosed PJI due to unrecognized suppressive therapy without thorough diagnostic work-up. This is an advantage in comparison to register data, which may not include all infected patients, because patients with antibiotic suppression or debridement might not be reported [1]. Another strength is the availability of a specialized interdisciplinary team caring for all patients with orthopedic infection. Thus, the risk for underreporting the early and mid-term infection risk of our patients is very low.

## Conclusions

The introduction of the DAA did not increase the risk of PJI. However a tendency for a higher fraction of polymicrobial and gramnegative PJI was observed in patients treated with the DAA.

## Abbreviations

ASA: American Society of Anesthesiologist
ASIS: Anterior superior iliac spine
BMI: Body mass index
CNS: Coagulase negative staphylococci
DAA: Direct anterior approach
DAIR: Debridement and implant retention
IDSA: Infections Disease Society on America
LAT: Lateral transgluteal approach
PJI: Periprosthetic joint infection
RA: Rheumatoid Arthritis
THA: Total hip arthroplasty
UPI: Unique patient identification number
